# Structural relaxation of ferroelectric phase in hard sodium lithium niobate solid solutions studied by solid-state NMR

**DOI:** 10.1038/s41598-025-15554-z

**Published:** 2025-08-21

**Authors:** Millena Logrado, Changhao Zhao, Hergen Breitzke, Jürgen Rödel, Gerd Buntkowsky

**Affiliations:** 1 Department of Chemistry, Eduard-Zintl Institute for Inorganic and Physical Chemistry, Technical University of Darmstadt, 64289 Darmstadt, Germany; 2 State Key Laboratory of Electrical Insulation and Power Equipment, School of Electrical Engineering, Xi’an Jiaotong University, Xi’an, 710049 Shaanxi PR China; 3 Division of Nonmetallic-Inorganic Materials, Department of Materials and Earth Sciences, Technical University of Darmstadt, 64289 Darmstadt, Germany

**Keywords:** Precipitates, LNN, NMR, LiNbO_3_, Materials science, Physics

## Abstract

Hard sodium lithium niobate (LNN) solid solutions offer a promising environmentally benign alternative to lead-based ferroelectric materials for electronic devices. A major challenge in their practical application is that their ferroelectric phase relaxes over a timescale ranging from weeks to years to an orthorhombic phase, limiting their long-term performance. In order to understand the structural changes in the relaxation process, high mechanical quality factor Li_*x*_Na_1−*x*_NbO_3_ solid-solutions were deliberately stored under ambient conditions for 24 months, without any specialized hermetic protection, to assess their stability over time. We utilized 1D and 2D ^23^Na solid-state Nuclear Magnetic Resonance (ssNMR) to investigate short-range structural changes in the 24-months-old aged and unaged Li_*x*_Na_1−*x*_NbO_3_ solid-solutions. NMR results reveal a system with phase-changes as a function of aging time and temperature. The samples exhibit a multiphase structure compromised of crystalline R and Q orthorhombic domains, along with two types of amorphous regions. A significant amount of ferroelectric phase persists in the ceramics after 24 months of exposure to ambient conditions. A structural model based on short-range order of sodium was suggested and agrees well with the lattice parameter of the freshly prepared samples.

**Supplementary Information:**

The online version contains supplementary material available at 10.1038/s41598-025-15554-z.

## Introduction

Lead-free piezoceramics are promising substitution materials for problematic lead-containing electric and electronic materials.^1–4^ Particularly, hard sodium lithium niobate (LNN) is non-hazardous, sustainable and environmentally benign. Depending on specific additives, it has excellent application potential in nonlinear photonics for high-density optical data storage, surface acoustic wave devices and piezoelectric devices^5,6^.

Pure LiNbO_3_ is a well-known material, reported to be in a rhombohedral phase R3c at room temperature, exhibiting a single ferroelectric-paraelectric phase transition at ca. 1210 °C.^7–9^ Pure NaNbO_3_, on the other hand, exhibits a complex set of seven distinct phase transitions over the range between − 100 °C and 640 °C.^10–12^ However, not just the rich phase transition diagram makes LNN an interesting material. Thermal treatments applied to LNN samples have demonstrated fundamentally new results on enhancing their mechanical quality factor.^13^ Recently, Zhao et al.^14,15^ succeeded in transferring precipitation techniques, commonly used in metallurgy, to tailor the properties of solid-solution ceramics. Mechanical losses are caused by the self-heating under high-frequency applications, ranging from few kHz to MHz, due to mobility of ferroelectric domain walls. Taking advantage of the strong temperature-dependent solubility of LiNbO_3_ in the NaNbO_3_ matrix, the precipitation of the Li-rich phase with the composition: *x* LiNbO_3_ - (1 - *x*) NaNbO_3_, where *x* = 0.18, clearly demonstrates a high efficiency in reducing mechanical losses.

A series of experimental studies – based on dielectric permittivity as a function of the temperature, X-ray diffraction (XRD), neutron diffraction (ND) and solid-state nuclear magnetic resonance spectroscopy (ssNMR) - has revealed the phase diagram of LNN.^16–18^ While the long-range order of LNN material has been extensively investigated and is better understood today, the local order on the short-range scale (0.2–0.5 nm) is still a source of debate. Due to the intrinsic local disorder characteristic of ceramics, the limited global understanding of the short-range order in LNN material has proven to be a significant challenge in comprehending its local structural arrangement of the lattice and its distortions. For example, studies using temperature-dependent dielectric permittivity, Raman spectroscopy and synchrotron XRD argued for a relatively narrow morphotropic phase boundary (MPB) at composition *x* = 0.12, whereas other studies using ^23^Na ssNMR, neutron powder diffraction (PND) and powder XRD testify to the complexity of local order-range by the multiphase coexistence regions over a much broader range of 0.15 < *x* < 0.9.^7,16,19,20^ Until now, at room temperature, LNN solid solution ceramics have been reported as exhibiting either one or the coexistence of the following phases: (a) ferroelectric orthorhombic Q phase, represented by the point group P21cm, (b) antiferroelectric orthorhombic P phase, represented by the point group Pbcm, and (c) ferroelectric rhombohedral R phase, represented by the point group R3c. The mechanism governing the phase change in this material has not yet been fully clarified.

Nuclear Magnetic Resonance has been a powerful technique to investigate the short-range order of materials characteristically disordered, such as glasses, local disorder in ceramics and phase transitions.^21–24^ Particularly, NMR was used to distinguish between ferroelectric orthorhombic Q phase and antiferroelectric orthorhombic P phase – distinction which is very unprecise using several other techniques.^25,26^

Studies^20^ combining ssNMR, PXRD, PND and ssNMR revealed spontaneous changes in the environment of (Li, Na)NbO_3_ upon the exposure of these samples to ambient conditions, revealing an unprompted change of the phase over time. Additionally, in the range of 0.08 < *x* < 0.15, the study demonstrates that both, the synthesis conditions and the storage-time at ambient conditions after synthesis, play a central role in the phases formed, revealing rapid phase relaxation processes occurring within a time frame of weeks to a year. For example, the sample with *x* = 0.15 yielded 45% of the Q phase after synthesis, but complete absence of this phase after one year. According to the same study, the phases in LNN in the above-mentioned range might be strongly dependent also on the cooling rate after sintering and the aging temperature.

Under this scenario, we carried out a careful investigation of the short-range order in the sodium environment of LNN samples reported by Zhao et al. [12] The samples, fabricated under different conditions of aging time and temperature, were exposed to ambient conditions for 24 months and just then, characterized by quantitative 1D and 2D^ 23^Na solid-state NMR spectroscopy. Our study reveals the coexistence of different sodium sites, including a significant amount of the ferroelectric phase. Furthermore, the structural changes that occur under the different aging treatments, which induce the formation of precipitates, led to the development of a structural model based on the short-range sodium environment. The results will contribute to the understanding of how the growth of the precipitates tunes the ferroelectric phase of the material.

## Results and discussion

**Fig. 1:**
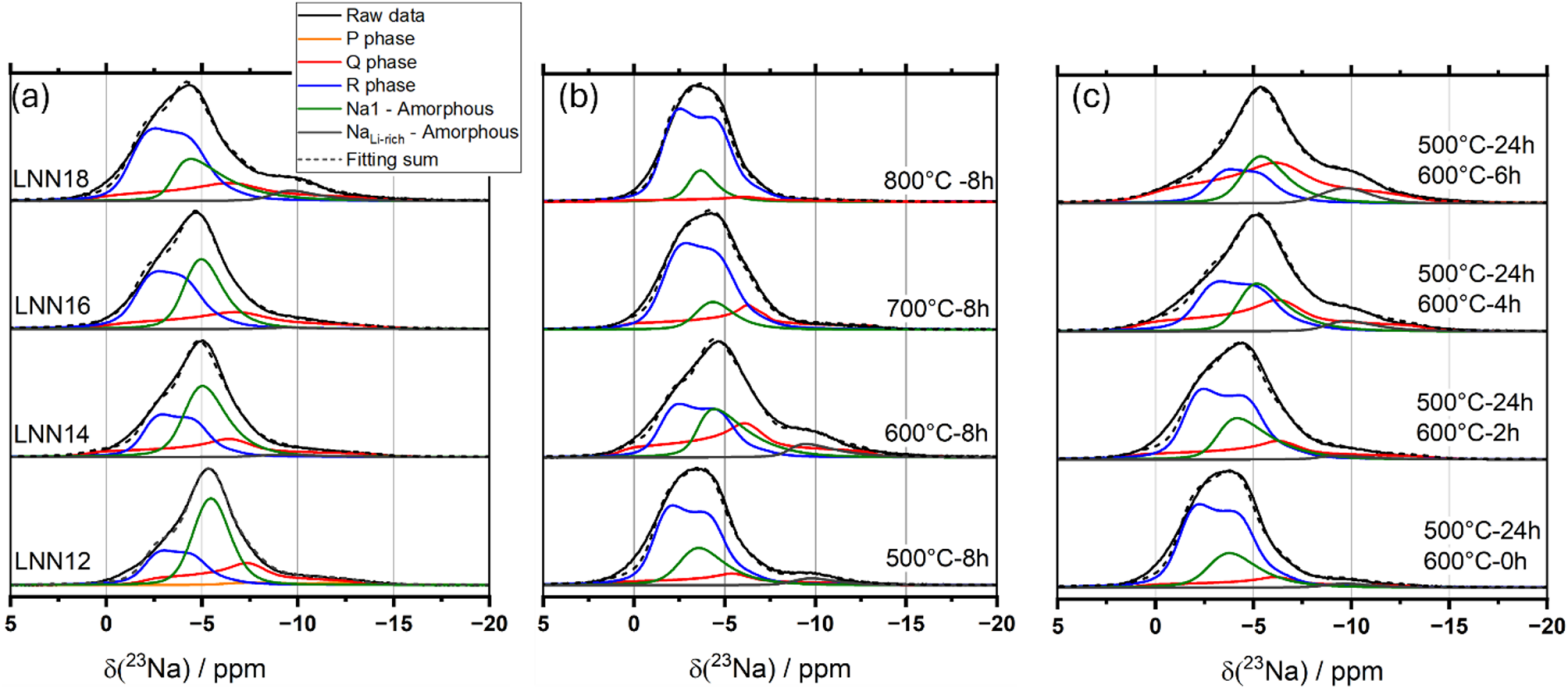
^23^Na MAS NMR spectra (red line), spectral simulation (dashed black line) and spectral decomposition spectral simulation of the samples: (**a**) LNNx, (**b**) LNN18 aged for 8 h at temperature T, with T є [500, 800] °C and, (**c**) LNN18 1st aged at 500 °C for 24 h and 2nd aged at 600 °C for t hours, with t є [0,6] h.

**Fig. 2:**
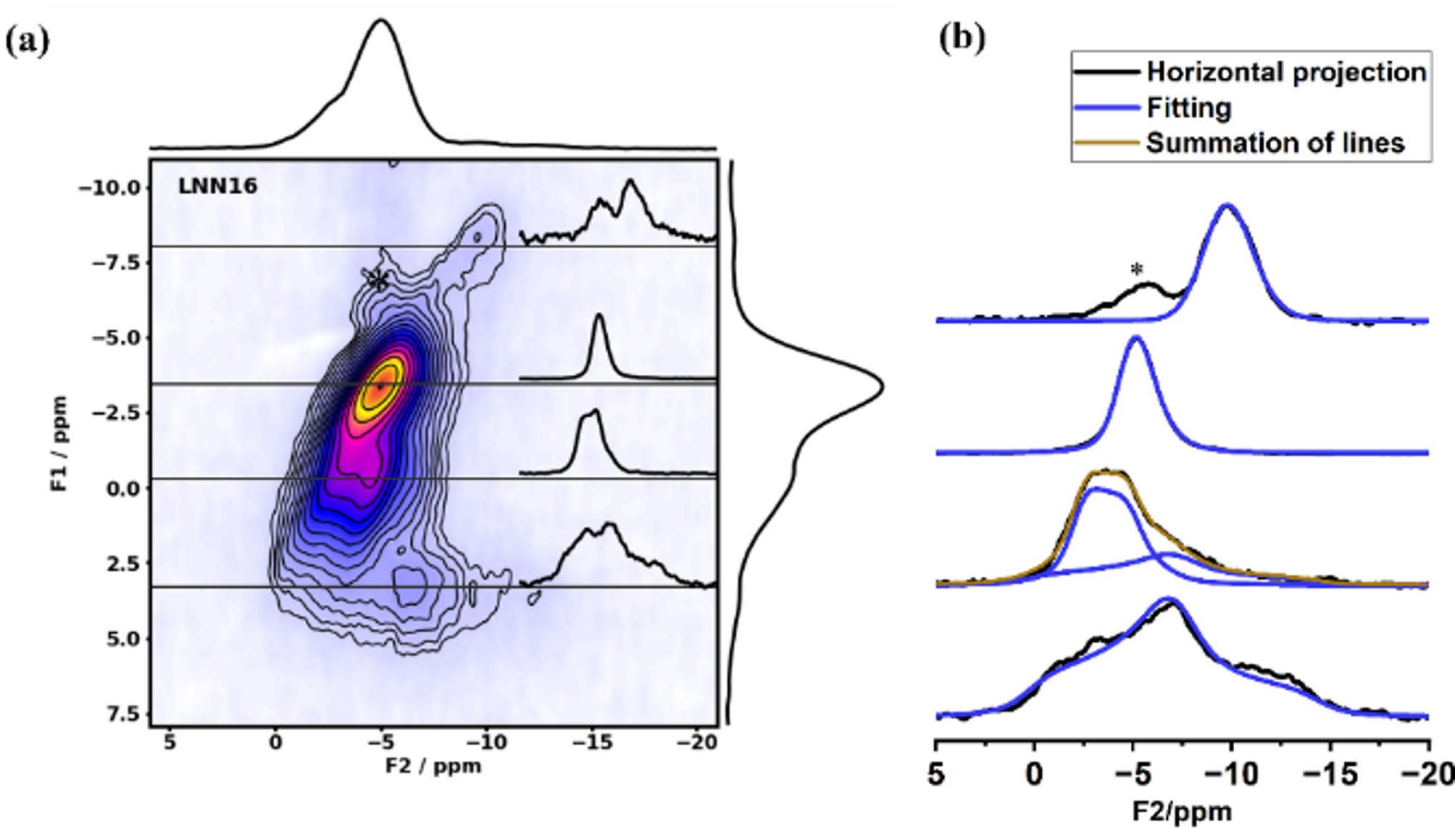
(**a**)^23^Na 3QMAS NMR spectra for the sample LNN18 aged at 500 °C for 24 h and at 600 °C for 6 h. The spectra on the top and on the right represent the projections onto the F2 and F1 axes, respectively. Above each horizontal line in the 3QMAS spectra box, the F2 projection corresponding to that same F1 shift position is displayed. F2 projection (solid black lines) illustrated in (**a**) is deconvoluted into one or more lines (solid blue lines) in (b). Asterisk symbol (*) corresponds to a smaller artifact in the 2D spectrum.

Figure 1 displays the spectra of ^23^Na MAS NMR for (a) the LNNx system with 0.12 < *x* < 0.18, (b) the LNN18 aged for 8 h at a temperature *T* ϵ [500,800] °C and (c) the LNN18 aged for 24 h at 500 °C and at 600 °C for a time *t* ϵ [0,6] h. All the spectra were measured under quantitative conditions. The lineshapes are dominated by second-order quadrupolar effects. To acquire high-resolution ^23^Na NMR spectra, a 3QMAS-NMR experiment was performed. Figure 2 (a) highlights an example of 2D 3QMAS performed in LNN18 aged for 24 h at 500 °C and at 600 °C for 6 h. The 2D 3QMAS graph displays the F2 axis versus the F1 axis and includes horizontal lines. These lines represent the F2 projections for each corresponding F1 value along the lines, indicating at least one distinct sodium environment for each projection. Different line shapes were used to fit each one of the distinct sodium sites present in the spectra, as illustrated by Fig. 2 (b). When a single symmetry dominates that particular sodium site, the quadrupolar asymmetry parameters were obtained in the simulation. On the other hand, for simulating the lines associated with the amorphous site, which can be understood as a distribution of distinct symmetries, the Czjzek model was used. In both cases, the isotropic chemical shift value and the quadrupolar product for each line were obtained by analyzing their center of gravity in both dimensions. Table S1 in the Supplementary Information file lists all the values used for the deconvolution of the single-pulse experiments, and Table S2 the parameters obtained from the ^23^Na 3QMAS spectra. Additionally, in the Supplemental Material,^27^ Figure S2, Figure S3 and Figure S4 display the complete set of ^23^Na 3QMAS of all the samples. Finally, the deconvolution of the single pulse experiment is depicted with gray lines in Fig. 1.

An attempt to deconvolute a one-pulse experiment of the LNNx systems into three or more separate lines without additional fitting constraints would be highly ambiguous and unreliable. Therefore, parameters extracted from 3QMAS were used as independent constraints in the spectral simulation of the one-pulse experiment. The complete set of aged and unaged LNNx samples were simulated as a superposition of contributions from five different sodium environments: (a) orthorhombic antiferroelectric P phase, (b) orthorhombic ferroelectric Q phase, represented by the P21cm group, (c) rhombohedral R ferroelectric phase, characterized by the group R3c, and (d) Am1 and (e) Am2, both representing the two amorphous sites found in LNN samples.

In the displacive P-Q phase transition, the atomic configurations of the two phases are very similar.^10^ The P and Q phase consist of by two distinct Na sites - named Na(1) and Na(2)^28,29^. The differentiation between P and Q phase is made based on the Na(1) site because Na(1) of the Q phase fingerprints a non-axial symmetry. In the Q phase, this sodium site occupies a more distorted environment, i.e. it has larger quadrupolar coupling compared to Na(2), caused by the tilting of the NbO_6_ octahedra, which breaks the axial symmetry and leads to a lower symmetry. This is in contrast with the P phase, where sodium is found in the center of the NaO₁₂ cage, occupying a symmetric environment. Thus, the formation of the Q phase can be clearly observed through the distinct set of NMR parameters of this site. The Na(2) sodium site is overlapping with the spectral region where the amorphous Am1 site is observed in these samples. This superposition was observed in both the one-pulse and the 3QMAS experiments. The signal deconvolution in the region of Na(2) and Am1, done in a free fit – i.e. with no parameter constraint - would compromise the lineshape analysis, resulting in an inaccurate estimation of the distribution of these two sites. To simplify that undesired freedom in deconvolution up to five curves, the Na(2) was ignored in the spectral analysis. This does not affect the qualitative result but might mean that the quantitative analysis of Q phase is underestimated in this work, as a fraction of the signal corresponding to Na(2) was not accounted for.

**Fig. 3:**
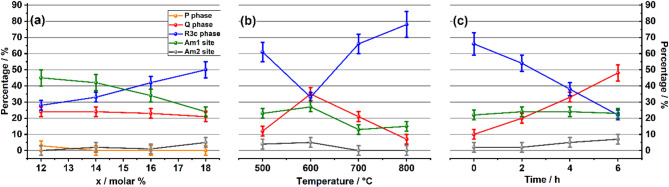
Amount of each phase present in (**a**) unaged LNNx, (**b**) 1st step aged LNN18 and (**c**) LNN18 aged in two steps. The letters Q, R represent, respectively, orthorhombic ferroelectric and rhombohedral phases.

Figure 3 illustrates the concentration of phases across the samples (a) unaged LNNx, (b) one-step aged LNN18, and (c) two-step aged LNN18. Figure 3 (a) demonstrates that, as lithium is gradually introduced into the sodium niobium matrix, the rhombohedral R3c phase steadily increases, while Am1 site systematically decreases and no consistent trend was observed for Am2 site, which is considered constant over the compositional range. Also, a small concentration of the P phase was found exclusively in unaged LNN12. Figure 3(b) reveals a clear non-linear trend in the distribution of the Q and R phases in the first step-aged samples. While Am1 and Am2 sites seem also to feature a similar tendency, pointing to a maximum at 600 °C, the evidence is not so clear as for the first two phases. Figure 3(c) illustrates that the two-step treatment − 500 °C for 24 h followed by 600 °C for x hours - progressively enhances the orthorhombic phase distribution during the second aging stage. While the amount of Na atoms found in Q phase and Am2 exhibit an increase, those found in R phase symmetry decrease steadily. Throughout the aging, the proportion of Am2 within the network remains constant.

- **unaged LNNx**: According to the phase diagrams for the LNNx material^16,20^, the detection of the antiferroelectric P phase in the LNN12 sample is a surprising finding, given its considerably high lithium content in the NN matrix. Typically, in this range, the material predominantly exhibits the R3c and Q phases. Even though the P phase is detectable, as clearly demonstrated by TQMAS (see Figure S2 for LNN12 in the Supplemental Material^27^), the percentage estimated by the simulation of single-pulse experiment indicates only a minor fraction of approximately 3%, which is close to the experimental error and highlights the difficulty in reliably quantifying this phase. This result agrees with previously reported phase diagrams^16,20^. Sample LNN12 verifies nearly equal amounts of the R and Q phases, suggesting that the morphotropic phase boundary (MPB) occurs near the *x* = 0.12 composition. Beyond this point, as lithium content continues to rise, the R phase becomes dominant. This outcome aligns with previous studies,^5,16,19,20^ which indicate that the phase diagrams often place the MPB close to *x* = 12 and a superposition of the R and Q phase for this compositional range.
- **One-step aged LNN18**: The maximum and minimum at 600 °C for, respectively, the Q and R phases highlight information around the nucleation and growth of the crystals. Assuming an optimal temperature for each, nucleation and crystal growth, lithium triggers the change in the sodium environment in this system.^13,14^ Given that the addition of lithium to the NN lattice disrupts its periodicity, the subtraction of lithium from the NN matrix – due to the formation of the LN rich phase – locally reverts the NN phase to a higher concentration of sodium.^14,30^ Owing to the fact that NMR is a volume-sensitive technique, neither the density nor the surface area of the precipitates affects the results illustrated in Fig. 3(b); instead, it is the total volume they occupy that plays a primary role. Therefore, the current experiment sheds light on the change in the sodium environment triggered by the formation of lithium niobate precipitates. The optimization of the volume of these precipitates, in turn, depends on the optimal temperature for nucleation and crystal growth. Under this perspective, we can infer that the optimal temperature for growing the crystal is ca. 600 °C, where the concentration of rhombohedral phase is minimum, and the concentration of desired orthorhombic ferroelectric phase is maximum. As the temperature for optimal nucleation should be below 600 °C, in this work, the value of 500 °C was assumed to be optimal.
- **Two-step aged LNN18**: The experiment suggests that the combination of two temperatures could further optimize precipitate growth. The system benefits from creating a high density of nuclei at lower temperature, reducing the diffusion length for the growth of the precipitates during the second heat treatment.

**Fig. 4:**
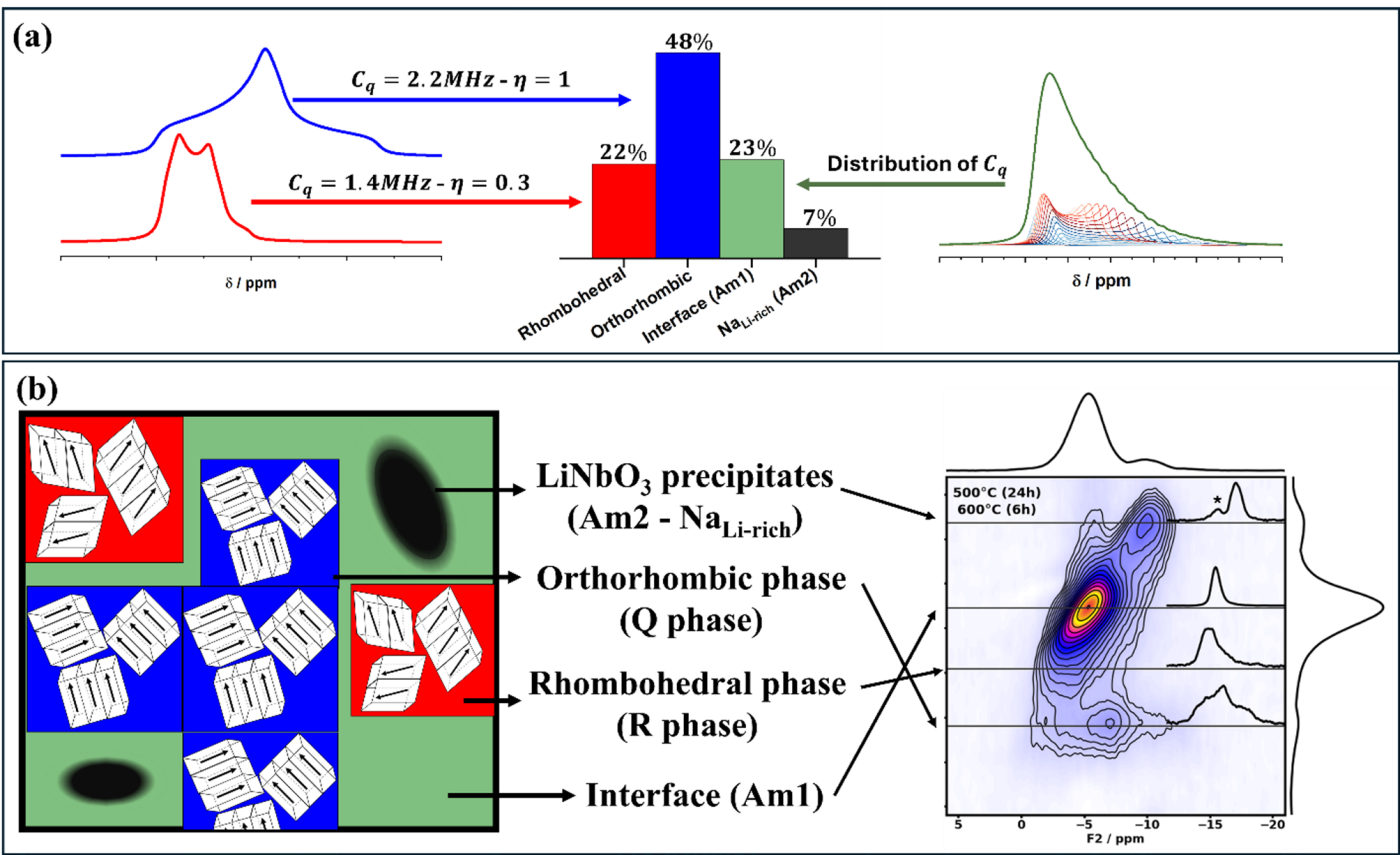
(**a**) On the right-side, the single symmetries are illustrated: rhombohedral, in red, and orthorhombic, in blue. On the left-side, the green signal illustrates the amorphous Am1 site, made of a distribution of distinct environments, namely, a distribution of quadrupolar coupling constant (C_q_). A similar distribution tail-shaped curve was used to simulate the Am2 site. The bar representation quantifies the proportion of each site for the sample LNN18 aged at 500 °C (24 h) in the first step and at 600 °C (6 h) in the second step. (**b**) The general interpretation of each of the ^23^Na signals. Using the same color notation, it illustrates the LiNbO_3_ precipitates, the orthorhombic Q phase, the rhombohedral R phase and the interface between these distinct sites. The proportion illustrated in the figure is equivalent to the sample LNN18 aged at 500 °C (24 h) in the first step and at 600 °C (6 h) in the second step.

The distinct sodium environments were summarized and interpreted in Fig. 4, which exemplifies the proportion and lineshape of each site using the sample LNN18 aged at 500 °C (24 h) and at 600 °C (6 h). Figure 4 (a) highlights those sites exhibiting a single symmetry - represented by specific quadrupolar parameters: the orthorhombic ferroelectric phases in blue and the rhombohedral phase in red. Additionally, it highlights the lineshapes resulting from a lack of specific symmetry, represented by a distribution of distinct symmetries: the green color (Am1) indicates the interface between different symmetries, while the black area (Am2) represents the LN phase. These distinct sodium environments were interpreted in Fig. 4 (b) with the same color notation. Each one of these phases and their interpretations are connected to the sites observed in 3QMAS.

By analyzing the proportion of ^23^Na at each site, we can interpret the structural configuration more clearly. In the fully aged sample, precipitates act as a sink for lithium. As a result, the lithium ions trapped in these precipitates are removed from the sodium-niobium matrix. This reduces the perturbation typically caused by an A-site with smaller cationic radius in an ABX_3_ perovskite. Consequently, the proportion of orthorhombic Q phase increases and the proportion of R phase reduces. Am2 reflects the amorphous character, as this site was interpreted as sodium trapped in the lithium rich phase. This hypothesis is based on what seems to be a correlation between the growth of precipitates and the increase in Am2 site proportion. Additionally, the Am1 phase is associated with the interface between distinct environments. At the interface - for example, between the rhombohedral and orthorhombic phases - unit cells might experience distributions of tilting axis directions, leading to distorted symmetry. In NMR, these distortions appear as a distribution of quadrupolar interaction, supporting the interpretation of the Am1 site as reflecting a strong amorphous character, particularly at the interface between different symmetries.

Note that this interpretation might overestimate the proportion of the Am1 site, as it could represent a combination of (i) an amorphous site and (ii) the Na(2) site of the Q phase, which might not be properly accounted for in the Q phase. Various attempts have been made to better deconvolute and understand the Am1 site. However, the similarity in quadrupolar parameters and isotropic chemical shifts made the separation of Na(2) site of the Q phase and the amorphous site unfeasible. Despite our efforts, our analysis of the high-resolution 3QMAS spectra did not indicate that the Am1 site was a combination of more than one site in any of the 12 distinct samples. Interestingly, a site with similar parameters as the Am1 was reported using 3QMAS in the investigation by Peel et al.^20^ in LNN samples with high content of lithium (*x* > 0.7). The 1D projection of the site in their study was not illustrated to allow a direct lineshape comparison with Am1 site. In their study, they suggest the hypothesis that this site might exhibit the R3 space group symmetry, derived by removal of c-glide. Until now, PXRD and DFT techniques were not able to probe their hypothesis. The inconclusive interpretation of the data was attributed to the disorder present. In the present work, we prefer to maintain the interpretation of an interface among phases, represented by a fully amorphous site, which appears consistent across all the samples. While the exact proportions of Am1 site may require refinement, the fundamental understanding and discussion of the phase behavior and its implications remains reliable.

According to the literature, an unprompted phase transition in (Li, Na)NbO_3_ samples was observed when the sample was exposed to ambient conditions.^20^ Based on the complete structural analysis provided by ssNMR, we investigated whether the initial insights on the long-range order parameters in the as-prepared samples, as published by Zhao et al.,^14^ indicate a correlation with the short-range order parameters reported by this study in 24-months-old samples. If no phase transition has occurred over the 24 months, the chemical shift values - which reflect the short-range order of sodium nuclei - are expected to correlate with the d_200_ parameter, as reported by the literature and explained bellow.^31–34^ This parameter measures the d-spacing of the (200)_NN_ plane in the sodium-niobium matrix, thereby indicating long-range order. It is expected that the systematic addition of a smaller cation, such as lithium, to the sodium-niobium lattice decreases the lattice parameter d_200_, bringing the Na-O bond length closer together. Chemical shifts of isotopes depend on the degree of magnetic shielding caused by the electronic cloud around the nuclei. If the Na-O bond lengths are shorter, the electronic cloud is more strongly attracted to the electronegative oxygen nuclei, leading to decreased shielding of the sodium nuclei. This deshielding of sodium results in greater exposure to the Zeeman field, leading to a higher chemical shift.

**Fig. 5:**
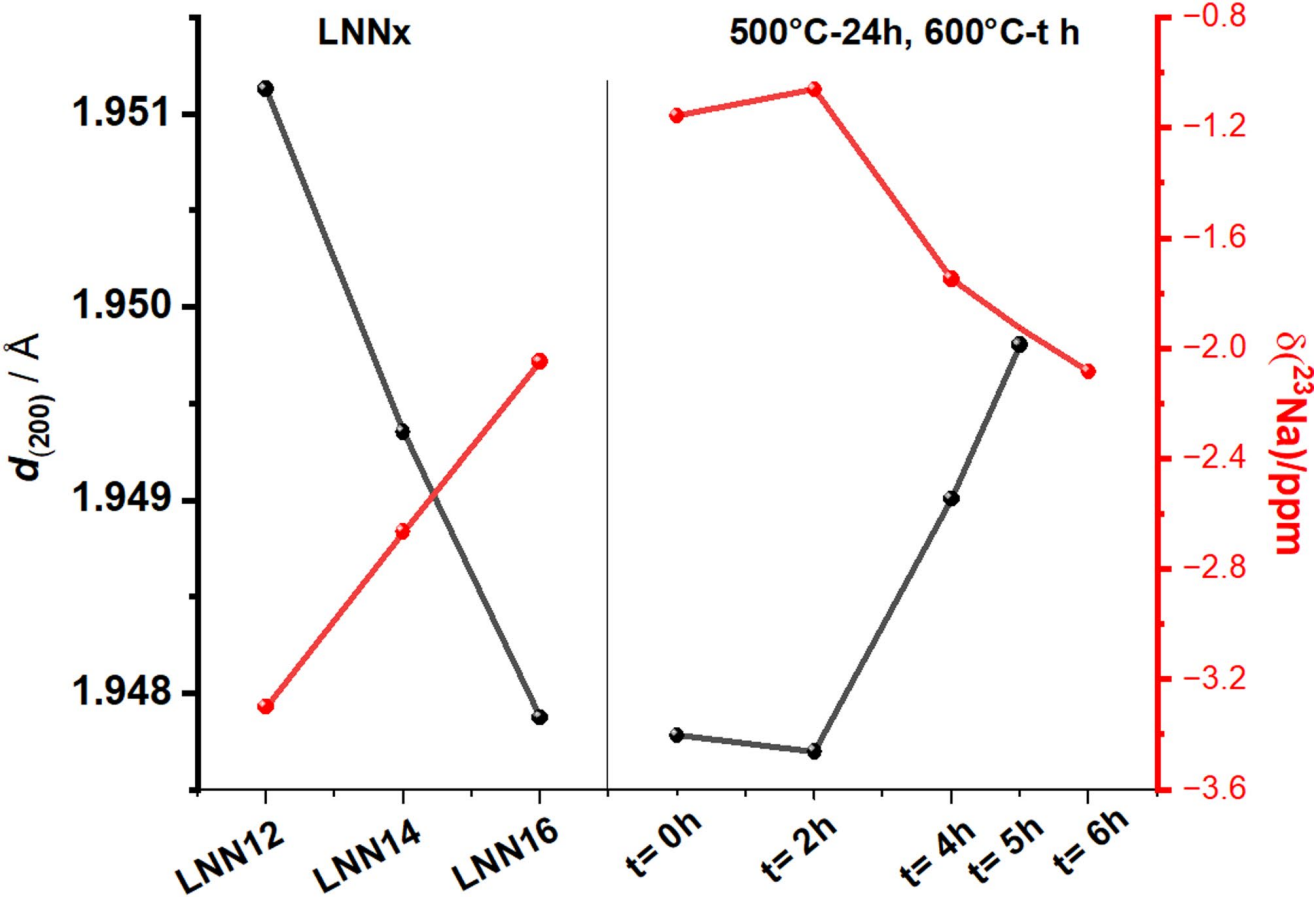
Trend of d-spacing of the (200)_NN_ plane of sodium-niobium matrix measured after the synthesis of the sample and the chemical shift of the samples measured 24 months after the sample fabrication in (left) LNNx samples and (right) LNN18 (second aged).^14^.

Figure 5 illustrates the behavior of the *d*-spacing of the (200) plane and the chemical shift across the unaged LNNx samples (left) and the LNN18 (2nd aged) samples (right). The left side of Fig. 5 demonstrates that the increase of the lithium fraction in the sample causes a decrease of the isotropic chemical shift and an increase in the d_200_ parameter. In Fig. 5, right, the LNN18 (2nd aged) samples feature a maximum chemical shift value and minimum d_200_ parameter at sample *t* = 2.

Statistics about the correlation between the d_200_ parameter and the chemical shift of the samples was calculated by expressing the lattice parameter as a function of the chemical shift, as provided in Figure S5 in the Supplemental Material.^27^ After fitting the dataset to a linear model, the R^2^ values of 0.998 and 0.995 were obtained for LNNx and LNN18 (second aged), respectively. Additional statistics related to the linear model can be found in Table S3. In both systems, the two variables appear to be strongly correlated according to this model.

These results, combined with the clear presence of Q phase in the material, indicate that the time delay between measurements - X-ray in the as-prepared samples and ssNMR 24 months later - did not significantly alter the phases in the samples. Additionally, these correlations suggest a plausible relationship between short- and long-range order.

Thefore the ^23^Na MAS NMR and 3QMAS clearly demonstrate the capability of solid-state NMR in revealing atomic-level insights of hard Li_*x*_Na_1−*x*_NbO_3_ (LNN) solid-solutions, both with and without distinct thermal treatments. Our research aimed to investigate the phase transition from the Q phase to the R phase over time by simply exposing the material to the ambient conditions (air). The analysis clearly shows that the local sodium environment features a coexistence between the R rhombohedral phase, the Q orthorhombic phases and two amorphous phases. 24 months after sample fabrication, the presence of a significant amount of the Q phase, mainly and expectedly in precipitate samples, suggests that the phase transition due to air exposure has not occurred. Additionally, a qualitative correlation was observed between the chemical shift and the lattice parameter. The correlation suggests that the expansion or contraction of the lattice – due to the addition or subtraction of lithium in the sodium niobium matrix – remains detectable through the solid-state NMR chemical shifts by reflecting the Na-O bond lengths. Furthermore, examining the short-range order was crucial in developing a structural model, particularly in ceramic materials where a local disorder is present. Understanding the structural changes over time is crucial for realistic applications. Overall, the structural model we developed for the hard LNN samples, the substantial presence of the Q phase, and the correlation between the short- and long-range order all indicate that these samples feature no signs of long-term instability, reinforcing precipitated LNN as a viable candidate to lead-based materials.

## Materials and methods

A broad range of solid solution ceramics with the composition Li_*x*_Na_1−*x*_NbO_3,_ were initially fabricated with *x* = 0.12, 0.14, 0.16 and 0.18, and are denoted by LNN12, LNN14, LNN16 and LNN18, respectively. All the samples were synthesized with a solid-state solution method with a sintering temperature of 1300 °C. The first set of LNN samples were quenched to avoid the formation of Li-rich phase (as LiNbO_3_ phase or LN phase).^14^ The second and third set served to focus on specific aging treatments [12], which cover both a nucleation and a growth stage of ensuing precipitates. To this end, samples were prepared using LNN18 as precursor and submitted to either one- or two-steps of thermal treatments, respectively. The second set of samples were aged for 8 h at 500 °C, 600 °C, 700 °C and 800 °C (one-step aging). The third set of samples was first aged for 24 h at 500 °C, followed by a second aging step at 600 °C for 0 h, 2 h, 4 h and 8 h (two-step aging). Both sets of samples are labeled as follows: temperature followed by the time of aging – e.g. “600°C(8 h)” when a single aging step was used or “500°C(24 h) − 600°C(6 h)” when two aging steps were used. The thermal treatment was applied to induce the formation of LiNbO_3_ precipitates, resulting in the lithium niobate phase (LN or lithium-rich phase) precipitating in a sodium niobate phase (NN or sodium-rich phase) matrix. Using the above scheme, a total of 12 different aging/composition variations have been considered in this work. More details about the sample fabrication are given in Zhao et al.^14^. Detailed investigations of the precipitate morphology by transmission electron microscopy have been provided before in same publication^12^.

According to Peel et al.,^20^ the LNN sample can form different phases depending on the cooling rate after the sample is synthesized, aging temperature, or due to the sample being left exposed to ambient air conditions. In the current study, the samples Li_*x*_Na_1−*x*_NbO_3_ were investigated 24 months after the sintering process. The samples were intentionally stored under ambient conditions, without hermetic sealing, to evaluate their long-term stability.

Solid-State ^23^Na NMR spectra were obtained at a Larmor frequency of 158.7 MHz on a Bruker AVANCE III 600 MHz spectrometer. A crushed sample, consisting of medium size aggregates of about 100s of microns, was packed into the 3.2 mm MAS rotors and spun at 20–25 kHz. The spectra were referenced to 1 M NaCl(aq) at 0 ppm. For single pulse measurements, a hard pulse was used with a B_1_-field strength corresponding to 60 kHz for NaCl. A flip angle of 30° and a recycle delay of 5*T_1_ corresponding to 60 s, were used in all the experiments to assure the full relaxation of the spins and the quantitative character of the spectra. Two-dimensional triple-quantum MAS (3QMAS our TQMAS) was used to eliminate the quadrupolar phase accumulated during the time evolution of triple quantum transitions by transferring the coherence to a single quantum transition. By refocusing the amplitude of the magnetization in a 2D experiment, the desired high resolution is achieved. 3QMAS spectra were recorded using z-filter pulse sequence.^35–37^ For the excitation/conversion pulses, the pulse duration and amplitudes were set to 4.8/1.8 us (flip angle of 330°/125°) employing a B_1_-field of 96 kHz calibrated via 0.1 M NaCl. The amplitude and duration of the third pulse of the 3QMAS were set to 15.6 kHz, respectively, 16 us measured on the sample. A recycle delay of 3.8 s was chosen for the 3QMAS experiments with the objective of shortening the experiment time. As illustrated by Figure S1 in the Supplemental Material^27^, the choice of delay recovery of 3.8 s, even distant from 5*T_1_ condition, does not affect the lineshape. Individual spectral lineshapes resolved by 3QMAS were simulated using SIMPSON and Ssnake software.^38,39^ Some spectral components revealed a lineshape characteristic of a distribution of chemical environments dominated by distribution of second order quadrupolar effect; these sites were simulated using a Czjzek model.^40^.

## Supplementary Information

Below is the link to the electronic supplementary material.

Supplementary Material 1

## Data Availability

Sequence data that support the findings of this study are available from the corresponding author and have been deposited in the link https://tudatalib.ulb.tu-darmstadt.de/handle/tudatalib/4626.
