# Supplementary material for: Structural relaxation of ferroelectric phase in hard sodium lithium niobate solid solutions studied by solid-state NMR
Source: Sci Rep. 2025 Aug 21;15:30755. doi: 10.1038/s41598-025-15554-z (PMC12370897; doi:10.1038/s41598-025-15554-z)
Supplement: Supplementary file 1 — Supplementary Material 1 [file 41598_2025_15554_MOESM1_ESM.docx]

**Supplemental Materials for**

**“**Structural relaxation of ferroelectric phase in hard sodium lithium niobate solid solutions studied by Solid-State NMR**”**

Millena Logrado, Changhao Zhao, Hergen Breitzke, Jürgen Rödel, Gerd Buntkowsky

**S1. Choice of delay recovery in ^23^Na MAS NMR**


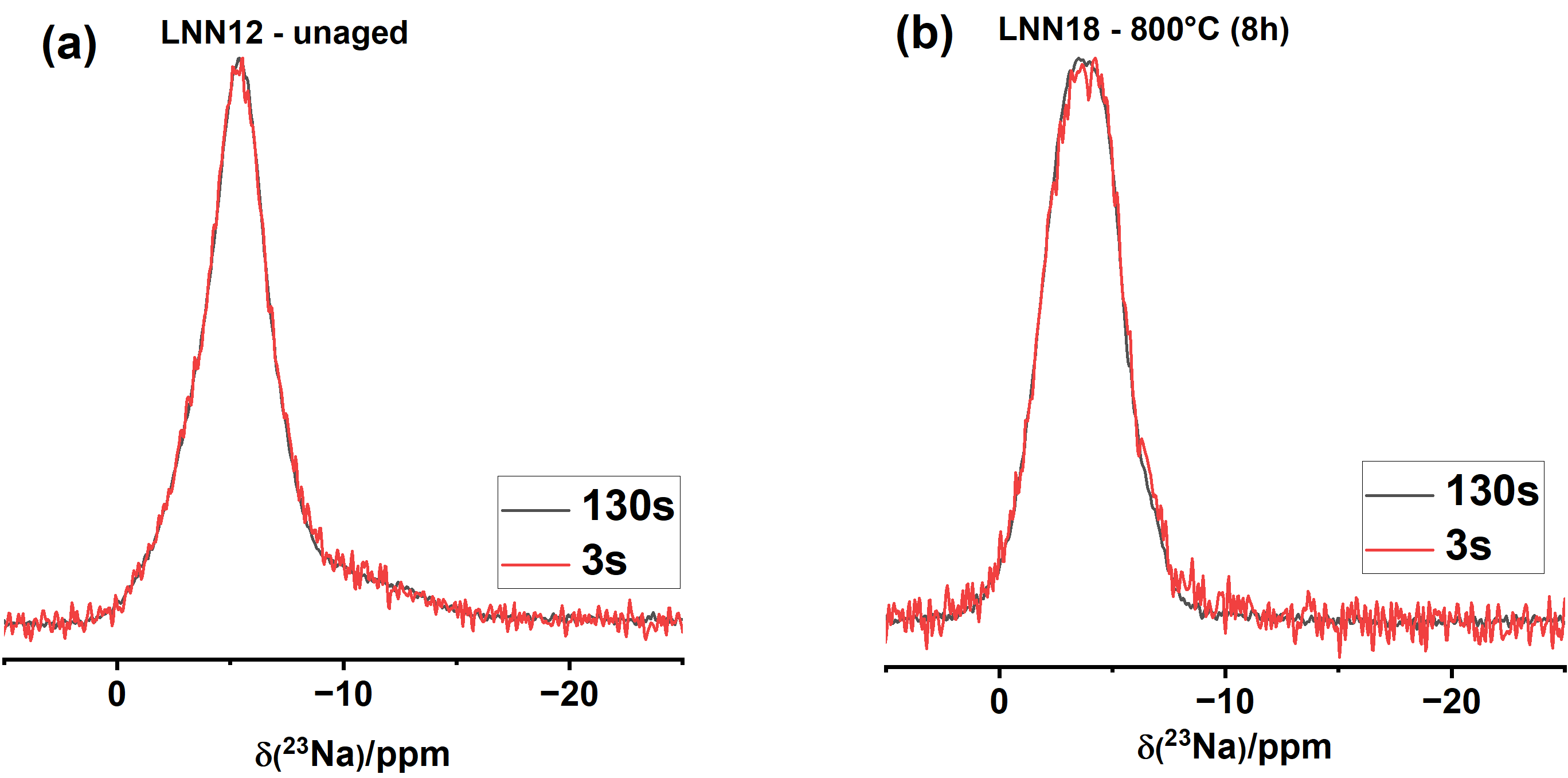


Figure S1: Normalized single pulse ^23^Na MAS under distinct relaxation delays for samples (a) LNN12 and (b) LNN18 aged for 8h at 800°C. A delay time of 3.8 s was used for MQMAS experiments.

**S2. ^23^Na 3QMAS spectra**


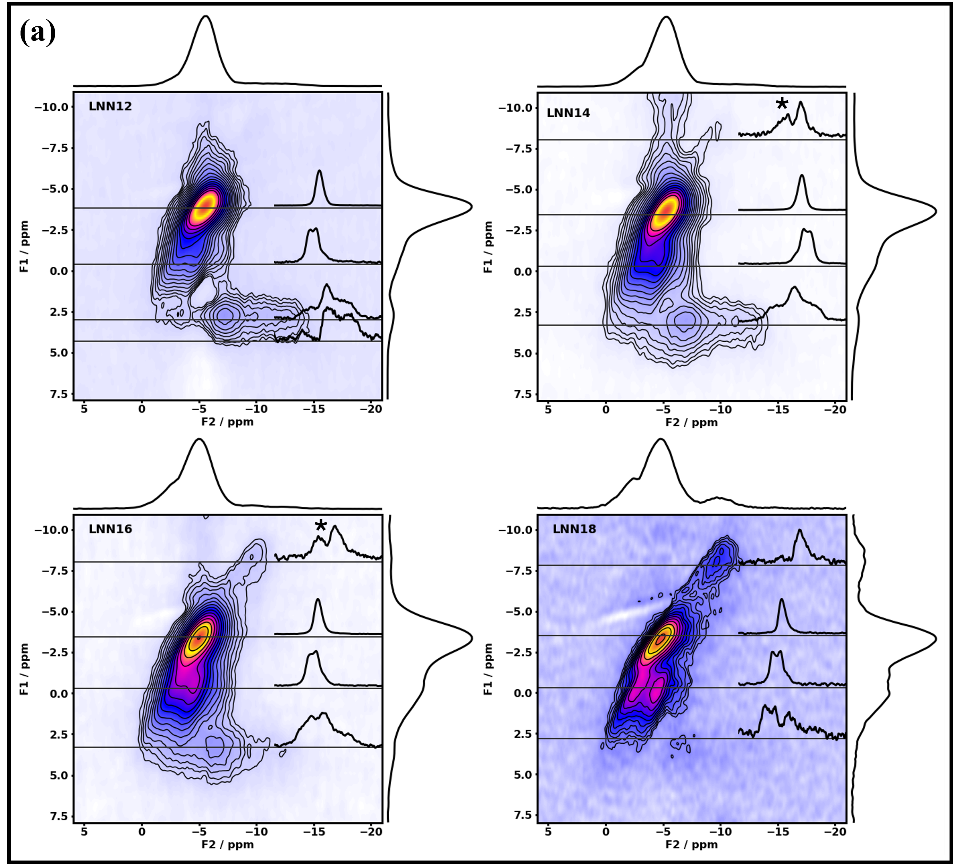


Figure S2: ^23^Na TQMAS results for LNNx system with x є [12, 18].


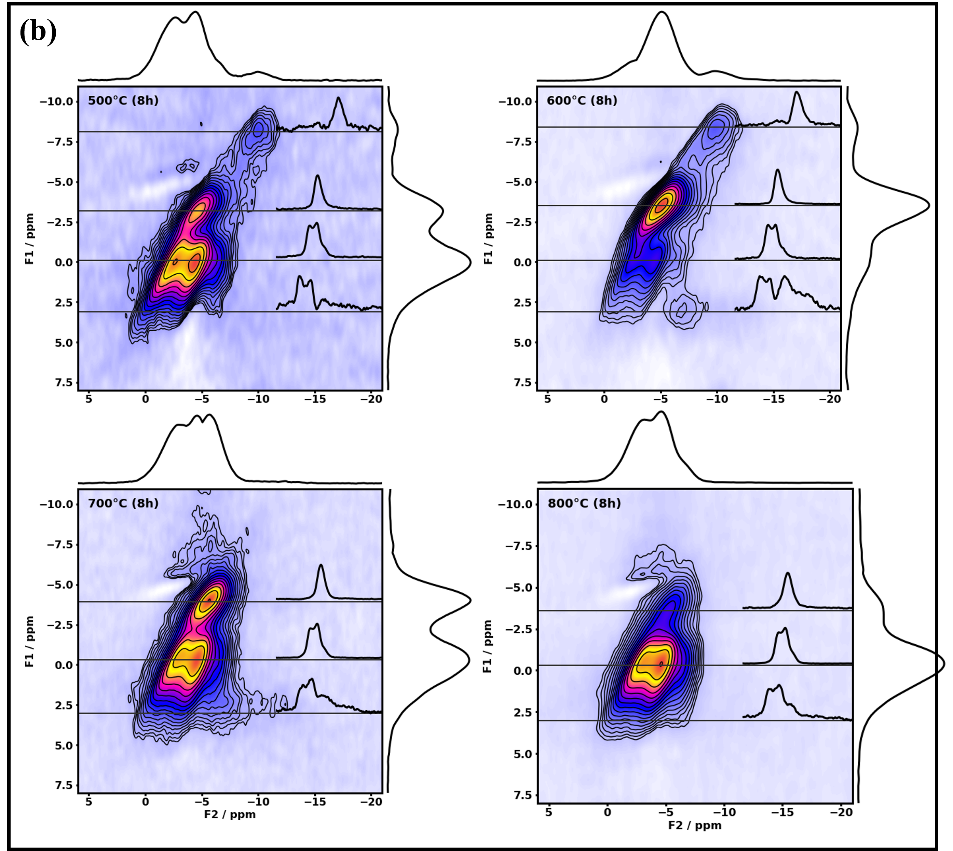


Figure S3: ^23^Na TQMAS results for LNN18 aged at a temperature T for 8 hours, with T є [500, 800] °C.


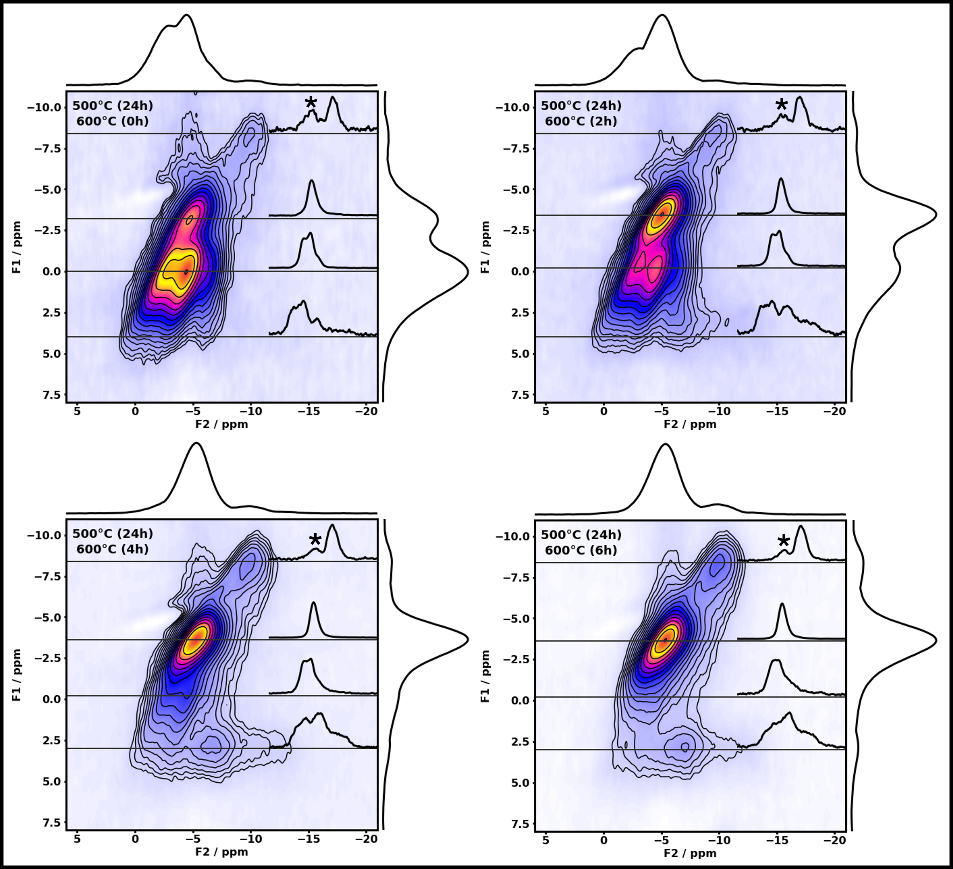


Figure S4: ^23^Na TQMAS results for LNN18 aged at 500 °C for 24 h and at 600 °C for a time t, where t є [0,6] hours.

**S3. Values used for the deconvolution of the single-pulse experiments and parameters obtained from the ^23^Na 3QMAS spectra**

Table S1: Quantitative parameters extracted from ^23^Na single pulse experiment. For each site or phase, the percentage, isotropic chemical shift (δ_0_), quadrupolar coupling constant (C_Q_) and asymmetry parameter (η_Q_) were measured using a single pulse experiment in a quantitative regime. The letters P, Q, R represent the orthorhombic antiferroelectric phase, the orthorhombic ferroelectric and the rhombohedral phases, respectively, while Am1 and Am2 represent two amorphous sites at higher and lower frequency, respectively. The error in the percentage was estimated as 10% of the corresponding percentage value. Values for absolute error less than 2 is unrealistic, therefore ± 2% is understood as the minimum error value.

| Sample | Site/Phase | Percentage / % | δ_0_ /  ±0.5 ppm | C_Q_ /  ±0.2 MHz | η_Q_ /  ±0.1 |
| --- | --- | --- | --- | --- | --- |
| LNN12 | P | 3 | -4.9 | 2.3 | 0.0 |
|  | Q | 24 | -1.9 | 2.0 | 1.0 |
|  | R | 28 | -1.6 | 1.4 | 0.0 |
|  | Am1 | 45 | -5.0 | 1.0 (σ=0.49) | - |
| LNN14 | Q | 24 | -1.2 | 2.31 | 1.0 |
|  | R | 33 | -1.5 | 1.49 | 0.0 |
|  | Am1 | 41 | -4.2 | 1.2 (σ=0.63) | - |
|  | Am2 | 2 | -8.0 | 1.3 (σ=0.70) | - |
| LNN16 | Q | 23 | 0.0 | 2.2 | 1.0 |
|  | R | 42 | -1.2 | 1.4 | 0.0 |
|  | Am1 | 34 | -4.3 | 1.0 (σ=0.50) | - |
|  | Am2 | 1 | -8.4 | 1.2 (σ=0.63) | - |
| LNN18 | Q | 21 | 0.3 | 2.2 | 1.0 |
|  | R | 50 | -0.8 (0.7) | 1.6 | 0.0 |
|  | Am1 | 34 | -3.4 | 1.3 (σ=0.66) | - |
|  | Am2 | 5 | -8.7 | 1.1 (σ=0.57) | - |
| LNN18  500°C  (8h) | Q | 12 | 0.3 | 2.2 | 1.0 |
|  | R | 61 | -0.8 (0.7) | 1.6 | 0.1 |
|  | Na1 | 23 | -3.7 | 1.3 (σ=0.54) | - |
|  | Am2 | 4 | -8.7 | 1.1 (σ=0.52) | - |
| LNN18  600°C(8h) | Q | 35 | 0.3 | 2.2 | 1.0 |
|  | R | 33 | -0.9 | 1.7 | 0.1 |
|  | Am1 | 27 | -3.4 | 1.2 (σ=0.65) | - |
|  | Am2 | 5 | -8.5 | 1.2 (σ=0.63) | - |
| LNN18  700°C(8h) | Q | 21 | 0.9 | 2.3 | 1.0 |
|  | R | 66 | -1.1 (0.7) | 1.6 | 0.0 |
|  | Am1 | 13 | -3.6 | 1.0 (σ=0.52) | - |
| LNN18  800°C(8h) | Q | 7 | 1.0 | 2.2 | 1.0 |
|  | R | 77 | -1.0 (0.5) | 1.6 | 0.0 |
|  | Am1 | 16 | -5.0 | 1.2 (σ=0.63) | - |
| LNN18  500°C(24h)  600°C(0h) | Q | 10 | 0.3 | 2.2 | 1.0 |
|  | R | 66 | -0.6 | 1.6 | 0.1 |
|  | Am1 | 22 | -2.8 | 1.1 (σ=0.54) | - |
|  | Am2 | 2 | -8.8 | 1.0 (σ=0.52) | - |
| LNN18  500°C(24h) 600°C(2h) | Q | 20 | 1.4 | 2.3 | 1.0 |
|  | R | 54 | -0.8 | 1.7 | 0.0 |
|  | Am1 | 24 | -3.1 | 1.2 (σ=0.63) | - |
|  | Am2 | 2 | -8.3 | 1.2 (σ=0.62) | - |
| LNN18  500°C(24h) 600°C(4h) | Q | 33 | 1.0 | 2.3 | 1.0 |
|  | R | 38 | -1.6 | 1.6 | 0.0 |
|  | Am1 | 24 | -4.3 | 1.1 (σ=0.57) | - |
|  | Am2 | 5 | -8.8 | 1.1 (σ=0.55) | - |
| LNN18  500°C(24h) 600°C(6h) | Q | 48 | 0.3 | 2.2 | 1.0 |
|  | R | 22 | -2.4 | 1.5 | 0.0 |
|  | Am1 | 23 | -4.6 | 1.0 (σ=0.50) | - |
|  | Am2 | 7 | -9.2 | 0.9 (σ=0.40) | - |

Table S2: Parameters extracted from ^23^Na 3QMAS experiment. For each site or phase, the isotropic chemical shift (δ_0_) and the quadrupolar product (P_Q_) were measured. The letters P, Q, R represent the orthorhombic antiferroelectric, orthorhombic ferroelectric and rhombohedral phases, respectively, while Am1 and Am2 represent two amorphous sites at higher and lower frequency, respectively. Asterisk mark (*) represents the sites with poor signal-to-noise ratio. Due that, their parameters were not able to be extracted accurately.

| Sample | Site/Phase | δ_0_/ ±0.5 ppm | P_Q_/ ±0.2 MHz |
| --- | --- | --- | --- |
| LNN12 | P | - | - |
|  | Q | -0.9 | 2.5 |
|  | R | -2.4 | 1.4 |
|  | Am1 | -4.3 | 1.0 |
| LNN18 | Q | -0.3 | 2.5 |
|  | R | -1.8 | 1.6 |
|  | Am1 | -3.9 | 1.1 |
|  | Am2 | -9.1 | 1.1 |
| LNN18  500°C (8h) | Q | * | * |
|  | R | -1.7 | 1.6 |
|  | Na1 | -4.1 | 1.1 |
|  | Am2 | -9.2 | 1.1 |
| LNN18  800°C(8h) | Q | * | * |
|  | R | -1.9 | 1.6 |
|  | Am1 | -4.5 | 1.0 |
| LNN18  500°C(24h)  600°C(0h) | Q | * | * |
|  | R | -1.6 | 1.6 |
|  | Am1 | -3.8 | 1.1 |
|  | Am2 | -8.9 | 1.2 |
| LNN18  500°C(24h) 600°C(6h) | Q | -0.6 | 2.5 |
|  | R | * | * |
|  | Am1 | -3.6 | 1.1 |
|  | Am2 | -9.0 | 1.1 |

**S4. Correlation between the lattice parameter and the ^23^Na chemical shift of the samples**


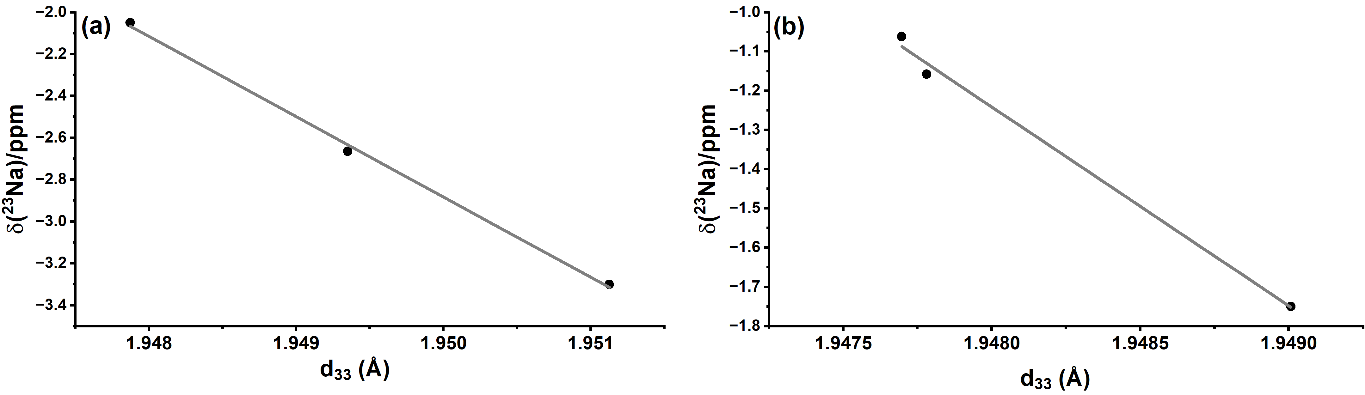


Figure S5: Lattice parameter (d_200_) as a function of the ^23^Na chemical shift for (a) LNNx samples, where x є [12,16], and (b) LNN18 for a time t, where t є [0,4] hours.

Table S3: F-value and R² from the linear models shown in Figure S5(a) and (b). An F-value greater than the critical value (Fc) indicates that the model is statistically significant at the chosen significance level. An R² value closer to 1 indicates that the model explains the variability in the data more effectively.

|  | Figure 6(a) | Figure 6(b) | F_c_=F_(1,1,S)_ |
| --- | --- | --- | --- |
| F-value | 539.01265 | 191.47748 | F_(1,1, 0.027)_ = 539.01265  F_(1,1,0.046)_ = 191.47748 |
| R^2^ | 0.998 | 0.995 | - |
